# Current Progress for Retrospective Identification of Nerve Agent Biomarkers in Biological Samples after Exposure

**DOI:** 10.3390/toxics10080439

**Published:** 2022-08-01

**Authors:** Jin Wang, Xiaogang Lu, Runli Gao, Chengxin Pei, Hongmei Wang

**Affiliations:** State Key Laboratory of NBC Protection for Civilian, Beijing 102205, China

**Keywords:** organophosphorus nerve agents, biomarkers, intact agents, degradation products, protein adducts, research methods

## Abstract

Organophosphorus neurotoxic agents (OPNAs) seriously damage the nervous system, inhibiting AChE activity and threatening human health and life. Timely and accurate detection of biomarkers in biomedical samples is an important means for identifying OPNA exposure, helping to recognize and clarify its characteristics and providing unambiguous forensic evidence for retrospective research. It is therefore necessary to summarize the varieties of biomarkers, recognize their various characteristics, and understand the principal research methods for these biomarkers in the retrospective detection of OPNA exposure. Common biomarkers include mainly intact agents, degradation products and protein adducts. Direct agent identification in basic experimental research was successfully applied to the detection of free OPNAs, however, this method is not applicable to actual biomedical samples because the high reactivity of OPNAs promotes rapid metabolism. Stepwise degradation products are important targets for retrospective research and are usually analyzed using a GC–MS, or an LC–MS system after derivatization. The smaller window of detection time requires that sampling be accomplished within 48 h, increasing the obstacles to determining OPNA exposure. For this reason, the focus of retrospective identification of OPNA exposure has shifted to protein adducts with a longer lifetime. Compared to the fluoride-induced reactivation method, which cannot be used for aged adducts, digestive peptide analysis is the more elegant method for detecting various adducts, identifying more active sites, exploring potential biomarkers and excavating characteristic ions. Retrospective identification of biomarkers after OPNA poisoning is of primary importance, providing unambiguous evidence for forensic analysis in actual cases and judgment of chemical accidents. At present, degradation products, the nonapeptide from BChE adducts and Y411 from human serum adducts are used successfully in actual cases of OPNA exposure. However, more potential biomarkers are still in the discovery stage, which may prove inconclusive. Therefore, there is an urgent need for research that screens biomarker candidates with high reactivity and good reliability from the potential candidates. In addition, mass spectrometry detection with high resolution and reactivity and an accurate data processing system in the scanning mode must also be further improved for the retrospective identification of unknown agents.

## 1. Introduction

**Figure 1:**
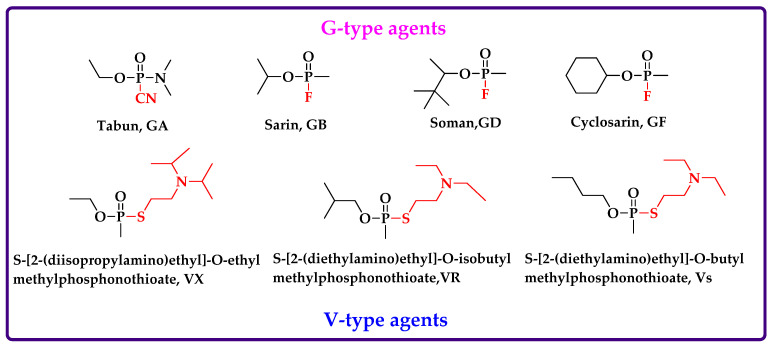
Common G-series or V-series OPNAs and their structure.

Organophosphorus nerve agents (OPNAs) first appeared in the middle of the 20th century, posing a great threat to human health, life and safety [1]. Traditional OPNAs, including G- and V-series agents, inhibit AChE activity by covalently binding to active site serine in acetylcholinesterase (AChE) [2,3] (common G-series or V-series OPNAs and their structures are shown in Figure 1). Inhibited AChE loses its ability to hydrolyze neurotransmitter acetylcholine (ACh), resulting in the continuous accumulation of ACh in synaptic junctions, and destroying the homeostasis in the nervous system, having a toxic effect on the human body [4,5,6]. OPNAs were once used on the battlefield as chemical warfare agents (CWAs) because they were easily synthesized and inexpensive [7,8]; in 1993, the Chemical Weapons Convention (CWC) prohibited the production, storage, use and transfer of such compounds [2,9]. Nevertheless, OPNAs have re-emerged in poisonings, assassinations, and terroristic attacks; the sarin (GB) gas attack in Tokyo in 1995 [10], GB positive appeared in the biological samples of victims in the Syrian war in 2013 [11,12], the assassination of Kim Jong-Nam in 2017 and the poisoning of Russian ex-spy Sergey Skripal and his daughter Yulia in 2018, are all reminders that the threat of OPNAs remains [13]. Therefore, it is essential to provide unambiguous evidence for OPNA exposure.

Upon entering the human body, OPNAs are distributed rapidly and are metabolized to exist eventually in vivo in three forms: intact compounds, degradation products and protein adducts. Colorimetric Ellman assay, based on residual AChE, or butyrylcholinesterase (BChE) activity, helps quickly and effectively identify OPNA poisoning [14,15]. However, the method can only approximate OPNA exposure due to its various shortcomings: it cannot determine the agent’s identity, or the different baseline of an individual’s AChE level and it cannot detect poisoning when inhibited ChE activity is less than 20%. Retrospective analysis by detecting OPNA biomarkers in biological samples has gradually become a significant means for investigating the use of chemical weapons. Biomarkers often take degradation products and protein adducts as biological targets because of the high reactivity of the intact agents resulting in fast degradation and transformation [16]. The degradation products and protein adducts in biomedical samples have longer half-lives than the intact poison. Unfortunately, within 48 hours after OPNA poisoning, about 90% of the degradation products circulating in biological fluids are excreted [17]. This very limited detection window makes it difficult for absentia analysts to provide a retrospective examination. Therefore, the focus of follow-up analysis has shifted to protein adducts with a longer half-life [18].

Protein adducts with longer half-lives (several weeks to months) are formed by a covalent connection between the phosphonyl group of OPNAs and active amino acid residues in target proteins. BChE with high reactivity, and albumin with high abundance in plasma or serum samples, are important targets in a retrospective analysis of OPNA exposure [19]. The nonapeptide in BChE adducts and Y411 in albumin adducts are recognized biomarkers for forensic analysis. According to the requirements of the Organization for the Prohibition of Chemical Weapons (OPCW), at least two biomarkers should be identified to confirm OPNA exposure in retrospective studies. For this reason, more and more potential biomarkers were excavated. Important evidence, such as characteristic ions in high abundance and modified peptides with good stability, are constantly being explored. All these potential biomarkers help to provide unambiguous evidence for forensic analysis.

## 2. Intact OPNAs

Identification of intact agents after exposure is usually performed with environmental samples—water, soil and air, or plant samples—but rarely in biological samples. High reactivity and poor stability of OPNA in the body promote rapid distribution and metabolism, making it very difficult to detect complete agents in blood or urine samples [20]. Common methods, such as LC–MS and GC–MS analysis, require extraction and purification of agents in samples via solid phase extraction (SPE) or liquid–liquid extraction (LLE), keeping steady by a rapid transition to organic solvents, such as isopropanol, acetonitrile and ethyl acetate, and long-time analysis, which are unsuitable for identifying intact poisons with short half-lives. Other methods were proposed to obtain evidence of OPNA exposure by direct agent identification. Erhard et al. [21] described a fast and specific direct competitive ELISA method, using monoclonal antibodies to make accurate measurements with simpler equipment, which omitted complicated extraction and sample preparation. The detection limit of 100ng GD/ml was reached. An enzyme-based microassay was also reported to determine residual OPNA levels in the blood; it directly detects GD based on the linear correlation between AChE activity and agent concentration after neutralizing a deproteinized blood sample, which then undergoes further centrifugation. The sensitivity of this method achieved 18–1820 pg per ml in a blood sample [22]. However, the premise for these methods is to obtain biological samples containing intact OPNA compounds. In actuality, where the absolute amount of OPNAs is low enough, only degradation products or adducts are detected in a biomedical sample—not residual agents. Additionally, the time difference in these various situations: a blocked exposure site, rushed emergency rescue or analysts staying away from the accident scene, may all miss the crucial sampling period in vivo containing intact agents [23]. This suggests that metabolites with longer half-lives—such as degradation products and protein adducts—may be important targets in retrospective determination after OPNA exposure.

## 3. Degradation Products

**Figure 2:**
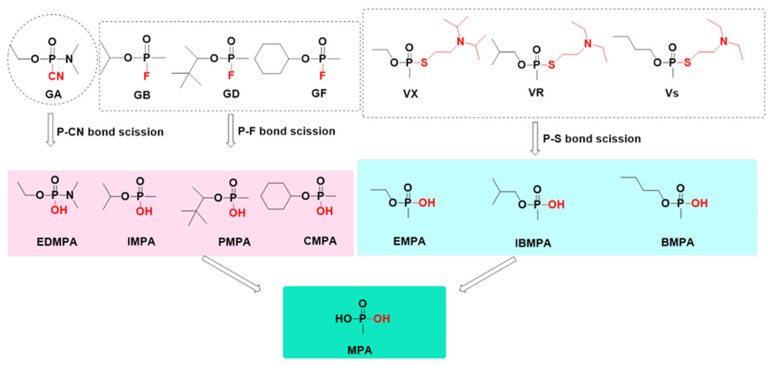
Stepwise degradation products and their structure.

Analytical chemists can identify OPNA exposure by detecting those degradation products caused by rapid distribution and degradation of OPNAs with high reactivity in the body. Products arising from OPNA degradation have a larger time window for detection than intact agents, helping to provide unambiguous forensic evidence for determining whether a subject was exposed to OPNAs [16]. The pathways of OPNA degradation rely mainly on enzymatic or spontaneous hydrolysis, and the products depend largely on the agents’ structural framework and chemical group composition, meaning that all OPNAs with common structural characteristics may perform similar degradation reactions and obtain similar—or even identical—products [24]. The OPNA degradation process consists of two steps. The initial reaction of OPNAs with water involves the formation of phosphonate monoester, which undergoes a second hydrolytic step to result in MPA, a final and mutual product arising from degradation. The greatest difference between degradation pathways of G- and V-series OPNAs lies in the primal hydrolysis reaction. In G-series OPNAs, the formation of phosphonate monoester is caused by P–F bond scission (or P–CN bond scission for GA). However, a similar hydrolysis reaction results from P–S bond scission in the structure of V-series OPNAs. Subsequently, the final reaction takes place slowly to obtain MPA. Stepwise degradation products and their structure are shown in Figure 2.

In related studies of retrospective identification of OPNA exposure, stepwise hydrolysates are used as biomarkers to provide more effective information. For example, Blanca et al. [17] utilized the LC–MS/MS system to analyze degradation products (IMPA) of GB in rabbit urine samples and found the eliminated pathways of IMPA. IMPA was excreted rapidly within 48 h after exposure, and the average half-life gradually increased after two days. Trimethylsilyl EMPA and MPA in human urine were identified and analyzed by GC–FPD analysis [24]. Detection limits were within 0.025 ppm for EMPA and 0.625 μM for MPA. Compared with the final hydrolysate MPA, rapid identification, chemical attribution and precise treatment can be promoted by the first degradation products, which to a great extent, retain the structural characteristics of OPNAs, except for the loss of specific groups caused by the breakage of unstable bonds. Degradation products derived directly from the intact OPNAs are important analytical targets for trace exploration.

Biological samples are usually identified by a GC–MS or LC–MS system. GC–MS analysis displays high sensitivity for biological samples, but the degradation products are polar and nonvolatile compounds, which cannot meet the requirements of GC–MS analysis for volatility and low polarity of the object component. Derivatization, which helps to improve the volatility and satisfy the adaptability of GC–MS, is an indispensable prerequisite in the preparation of biological samples. Young et al. [25] described a novel method to evaluate six agents (GA, GB, GD, GF, VX and VR) and silylated degradation products (EDPA, IMPA, PMPA, CMPA EMPA and MMPA) using the GC–MS/MS system, with a mid-polarity column. They found that this method simultaneously detected the agents’ precursors and metabolites in plasma to identify OPNA exposure. Commonly used derivation methods of degradation products usually include silylation and alkylation, which help to form a derivative by connecting silyl or alkyl groups to the polar analyte and converting analyte, making it suitable for GC–MS detection and analysis [26,27]. Other derivatization reagents, such as pentafluorobenzyl bromide and 2-(bromomethyl)- naphthalene, are also widely used in degradation product detection [28]. The derivatized hydrolysate can be directly used for GC–MS analysis. Unfortunately, prior derivatization to volatile compounds (often complicated and time-consuming) may increase the difficulty of analysis, producing more by-product formation, strong background signals, and ion source pollution in a GC–MS system.

Compared to GC–MS analysis, the LC–MS method eliminates derivatization because the degradation products are polar compounds and were widely utilized because of their high sensitivity to hydrophilic compounds. The LC–MS system was used for the analysis of various biological samples: blood, urine and other matrices with the function of protecting biomarkers, including hair and nails. A dried blood spot (DBS) on a filter paper can also be identified using LC–MS technology [29]. However, LC–MS analysis is not a detection method that pinpoints all hydrolysates; for some special compounds, derivatization technology is still indispensable. For example, MPA with high polarity and low molecular weight have insufficient retention time in the reversed-phase column; corresponding peaks significantly overlap with other components; low MS signals and high energy is required in the dissociation induced by collision, reducing the sensitivity of LC–MS analysis [30]. For this reason, prior pentafluoroben zylation helps add hydrophobic groups to reverse the short retention time. 

Other analytical methods also detect degradation products, such as ion chromatography (IC) analysis [31]. Katagi et al. [32] simultaneously explored several alkyl phosphoric acids via IC analysis after sample preparation using an Ag^+^-form cation-exchange resin cartridge. The authors found that the detection limits of MPA, EMPA and IPMPA were 40 ng/mL and the limit of PMPA was 80 ng/mL. As for special MPA, LC–MS analysis with derivatization can be replaced by IC analysis. MPA with a large molecular weight is suitable for IC analysis equipped with an indirect photometric detection (IPD) technique [32]. This analysis method omits complicated derivatization, prolongs retention time in the column, avoids transferring the sample to the organic solvent and reduces interference components. Son et al. [33] also reported the determination of EMPA, IMPA and PMPA in a human urine sample using the TOF–MS system with a UV femtosecond laser, emitting at 267 nm for efficient resonance-enhanced two-photon ionization (RE2PI). The derived analyte, obtained by using 2-(bromomethyl) naphthalene, added a 2-bromomethyl group to the -OH group of alkyl phosphoric acid and included a naphthalene chromophore to improve RE2PI efficiency [33].

## 4. Protein Adduct

The formation of protein adducts is based on the phosphonyl group in OPNAs structure covalent connection with the active site in the amino acid sequence of the target protein. In short, the inhibition of OPNAs on target proteins is caused by agent molecules attacking the enzyme and inserting active side chains in the amino acid sequence of one or several active sites. Retrospective research has determined that, compared to intact compounds and their degradation products, protein adducts with a longer half-life are more suitable as potential biomarkers, since their lifetime in the body is consistent with the life cycle of proteins. AChE was once considered to be the only target protein of agents, until BChE with high reactivity, albumin with high concentration and other proteins, such as ubiquitin, keratin and transferrin in blood samples, were reported one after another [34]. BChE adduct—a biomarker for exposure—is the first choice for forensic analysis, compared to the hard-to-obtain AChE adduct [35]. BChE adducts are often utilized as research targets to permit verification of the OPNAs exposure of low concentration and the high reactivity of BChE promotes preferential reaction with OPNAs [36]. Unfortunately, ChE adducts tend to age, reducing the possibility of clearly distinguishing OPNA identity [37]. Additionally, albumin with high concentrations in serum or plasma forms stable OPNA adducts, which are not easily denatured [38]. Thus, albumin adduct is also an important target for forensic verification after OPNA exposure. Other trace proteins in the blood were also examined in the retrospective study, but sparse valid data cannot offer relevant forensic evidence. Indeed, biomacromolecules, such as AChE, BChE and albumin, phosphonylation by OPNAs cause only slight changes in molecular weight, which are very difficult to observe directly by mass spectrometry. Therefore, two analytical methods were developed to help retrospective detection of protein adducts: fluoride-induced reactivation and digestive peptides analysis via enzymatic hydrolysis.

### 4.1. Fluoride-Induced Reactivation

Fluoride-induced reactivation is an elegant method for obtaining the origin and physicochemical properties of OPNAs in retrospective research after OPNA exposure. This method is based on the nucleophilic attack of fluoride ions on the phosphonyl moiety, in which fluoride forms a P–F bond with the phosphonyl moiety released from the target protein in serum or plasma samples by incubation with concentrated fluoride under acidic conditions [39]. Compared with trace AChE (~3 nM) bound to red blood cells, BChE (~80 nM) with high abundance in plasma is an important target for fluoride reactivation. In biomedical samples from terroristic attacks in Tokyo and Matsumoto, GB positive was identified by this methodology [40]. Unfortunately, when regenerated compounds are identical to the original form, it is impossible to determine whether agents originated freely or from regeneration. A separate assay, without incubation of excess fluoride, helps provide reliable evidence for the existence of free OPNAs [41]. Additionally, the rapid aging of OPNAs-BChE adducts may hinder the regeneration of fluorophosphate caused by electrostatic repulsion of F-ions and anionic phosphonic acid residue [42]. 

Commonly used methods for the analysis of regenerated fluorophosphates are GC or GC–MS analysis after the incubation of concentrated fluoride in biological samples at an acidic PH, SPE with ethyl acetate, and solvent removal via dry ice/acetone. Generally, the known regenerated compound can be analyzed in the mode with the highest reactivity. For example, Adams et al. [39] determined the regenerative agents in blood and tissues from guinea pigs exposed to GB and GD using the GC–MS system with selected ion monitoring (SIM) [39]. Both detection limits were as low as 0.5 ng per mL. However, unknown regenerated fluoride in actual biomedical samples must operate in the scanning mode of MS, which greatly reduces sensitivity. For this reason, van der Meer et al. [42] proposed a comprehensive GC (GC × GC)-MS which allowed efficient detection of trace analytes, with good selectivity in high sensitivity. Active sites in red blood cells (RBC) are also greater than those of BChE in plasma, which means that RBC tends to be an important matrix for studying regenerative compounds. McGuire et al. [43] described a sensitive method for detecting regenerated ethyl methylphos-phonofluoridate (VX-G) in red blood cells using isotope-dilution GC–MS/MS analysis with a triple-quadrupole. The detection limit reached <1 pg on the column, significantly lower than 10.5pg agents using GC with a flame-photometric detector and 3 pg agents via GC–HRMS analysis in human serum samples in the same type of research for regenerated VX-G.

In addition to the agents bound to AChE in red blood cells and BChE agents in serum or plasma, albumin adducts in plasma samples can also be analyzed by fluoride regeneration methods. Li et al. [44] found that fluorotabun, regenerated from the GA-albumin adducts in human serum, was identified by GC–MS/MS and displayed great linearity in the concentration range from 0.02 to 100.0 ng/mL. Furthermore, the LC–MS/MS system was reported to detect regenerated GB in biological samples. GB-2-DMAMP was determined by LC–ESI–MS/MS (MRM) analysis after derivatization with 2-[(dimethylamino) methyl]phenol (2-DMAMP) [45]. The limit of quantitation reached 5pg/ml. This analytical approach seems more complicated, but sensitivity and specificity were superior to those from the existing GC- and GC–MS methods.

### 4.2. Digestive Peptides Analysis

Identifying modified peptides from protein adducts is the most promising method for OPNA exposure. It not only reduces the difficulty of identifying small mass changes in biomacromolecules labeled by OPNAs using mass spectrometry but also avoids confusion between regenerated and free agents in fluoride-induced reactivation research when regenerated compounds are identical to the original form. Meanwhile, aged protein adducts can also be accurately detected and identified. The biological analysis procedure for digested peptides includes: extracting and purifying target protein from biological samples, hydrolyzing the extracted protein with protease to obtain peptide segments, and analyzing modified and unmodified peptides using the LC–MS system with mass selectivity [18]. 

Proteases are commonly used in enzymatic hydrolysis of adducts including pepsin, trypsin and pronase. Different proteases have different cleavage sites on amino acid sequences, and the peptide fragments obtained are also different [46]. Some peptides are obtained by pepsin digestion and are usually shorter than those obtained by trypsin hydrolysis because pepsin has more restriction sites than trypsin. However, there is no specific cleavage site for pronase, and the peptide segments obtained may be more random. The enzymolysis procedures of different proteases also vary. For example, pepsin is used under acidic conditions, while trypsin is used in weak alkaline conditions, and the operational procedure is more complicated than pepsin [47].

The most common analysis methods for modified peptides are the MALDI-TOF MS assay and the LC–ESI MS/MS system, both have high sensitivity and reproducibility [48,49]. However, multi-reaction monitoring (MRM) with the highest sensitivity is applicable only to known agents. For these reasons, the LC–MS/HRMS method was proposed to identify novel dipeptides from disulfide adducts and nLC–Q–Orbitrap–MS was used to explore potential biomarkers in the soman–albumin adduct [50,51]. These methods displayed high selectivity, good reliability and can quickly and accurately obtain MS data from samples.

The aim of analyzing digestive peptides from protein adducts is to obtain biomarkers that can be used to determine OPNA exposure. The method determines exposure to OPNAs by analyzing mass changes in the peptide digested by protease, and identifies the identity of the labeled agent by analyzing the increased molecular weight in the modified peptide and the characteristic ion peak in the fragmented ion spectrum, and clarifies the active sites in the target protein. At present, the nonapeptide from the BChE adduct and Y411 from the albumin adduct are recognized biomarkers for forensic verification and were used in actual case situations. According to the requirements of OPCW, at least two biomarkers must be identified to confirm OPNA exposure. Therefore, more potential biomarkers and modified peptides are continuously excavated, and characteristic productions are constantly discovered.

#### 4.2.1. AChE Adduct

As the inhibitors of AChE activity, OPNAs entering the organism were subjected to attack by the nucleophile of active site serines, resulting in the formation of OPNAs-AChE adducts, accompanied by the exfoliation of the departing group [1,7]. Thus, phosphonylated serine is mainly responsible for OPNA poisoning. AChE exists mostly in the synapse of the central nervous system, as well as a small amount of AChE, which is bound to the membrane of red blood cells in the blood. Compared with AChE in the synaptic cleft, AChE bound to an erythrocyte membrane seems easier to obtain. To our knowledge, only two articles describe the research methods of AChE adducts bound to an erythrocyte membrane: Dafferner et al. [52] proposed the concept of *no-ghost RBC AChE*, which is prepared by solubilizing AChE bound to a membrane from frozen RBC, using 1% triton x-100 in PBS and 0.1% azide. The LC–MS/MS system analyzed digested peptides of inhibited RBC AChE utilizing pepsin after immunopurification with anti-AChE sepharose beads. This method identified the classic MS/MS spectrum of the active peptide phosphonylated by aged GD, the parent ion (874.35 Da) of the modified peptide (consisting of peptide FGES*AGAAS, the remaining mass of GD (after losing the pinacolyl group), and a proton). The four most obvious peaks in the fragmented ion spectrum were at 874.36 Da (parent ion), 778.35 Da (losing methylphosphonate and a molecule of water via β-elimination from the parent ion), 673.31 Da (losing C-terminal serine), and 602.27 Da (losing C-terminal alanine and serine), respectively. A year later, the research group proposed an alternative enrichment strategy, in which immunopurification for RBC AChE was replaced by a Hupresin affinity column, resulting in a reduction in the amount of frozen RBC [53] used. The disadvantage of these methods lies in the requirement for a large volume of frozen RBC, even if the enrichment method for inhibited AChE is changed.

#### 4.2.2. BChE Adduct

The detection of the BChE adduct is the dominant means to retrospectively verify OPNA exposure. BChE adducts are easily produced because of the high reactivity of BChE to OPNAs and are detected in plasma or serum samples within 16 days after exposure [54]. Belonging to serine hydrolase, BChE has similar structural characteristics and a highly homologous amino acid sequence to AChE, resulting in the same formation mode of OPNAs-BChE adduct as that of AChE [37]. The difference is that the OPNAs-BChE adduct is safe for organisms. BChE can act as an endogenous stoichiometric scavenger to remove harmful agents through neutralization, or as prophylactic bioscavengers to prevent toxicity [55]. It was reported that the reaction rate of BChE to OPNAs is faster than that of AChE. However, there is insufficient BChE in plasma (4 μg/mL) to resist high concentrations of OPNAs, so exogenous BChE is developed as an antidote [56].

Specific phosphonylated nonapeptides from inhibited BChE obtained by pepsin digestion can be used as biomarkers for OPNA exposure and they were applied to serum samples from victims of the Tokyo terrorist attack in 1995 [57]. This analysis method is based mainly on characteristic fragment ion peaks of nonapeptide FGES_198_AGAAS, detected by an electrospray LC/tandem MS system. Four product ions with high peak values in the MS spectrum were at 916.4 Da (parent ion), 778.4 Da (losing IMPA from parent ion), 673.3 Da (b_8_^+^: losing C-terminal serine), and 602.27 Da (b_7_^+^: losing alanine and serine from C-terminal), respectively [58]. These stable product ions were confirmed repeatedly in biological experiments, providing relevant information about GB exposure [57,58,59,60]. The various peptides from BChE adducts are produced by digestion of different proteases, and the product ions shown in the MS spectrum are also different. Tsuge et al. [61] used ESI–MS to explore the peptide digested by trypsin from a human sample after purification by the SDS-PAGE system. An obtained peptide with an active center at S226 covers 28 amino acids and a molecular weight of 2989 Da, which is too large to offer reliable signals. However, analyzable peptides GESAGAASVSL (m/z 948.46) were obtained by chymotryptic digestion for inhibited BChE. The common product ions from GB-, VX-, GD- and aged GD adduct were found at 930.5, 799.4, 712.4, 613.3, 526.2 and 455.2.

These characteristic product ions—with specificity like fingerprints—help to accurately confirm the exposure of certain agents or certain types of OPNAs. Thus, iconic product ions are usually used for qualitative screening of OPNA exposure, applicable to any aged or unaged OPNA adducts. The biggest challenge of aged adducts losing specific O-alkyl phosphate is that they cannot specifically determine the identity of OPNAs. However, characteristic ions from aged adducts can help provide forensic evidence of OPNA exposure. Aged BChE adducts with OPNAs, such as GB, GD, GF, VX and VR, contain common methyl phosphonate MeP-BChE, which undergoes UHPLC–MS/MS analysis after digestion by pepsin to obtain product ions m/z 778.3 and 673.3 [62]. Other aged adducts such as propyl phosphonate ExP-, ethyl phosphonate EtP- and propyl phosphonate P-BChE, also produce these two characteristic product ions; the difference lies only in the different precursor ions, which can help to accurately classify OPNA adducts. In addition, John et al [63] proposed a novel propionylated nonapeptide biomarker after derivatization by adding propionic anhydride to phenylalanine residue (F_195_) from an N-terminal. This highly sensitive method was based on a µLC–ESI MS/MS system. The authors believed that propionylated nonapeptide could be used as a candidate biomarker for forensic verification. 

#### 4.2.3. Albumin Adduct

To facilitate ease of sampling, highly abundant albumin in plasma is an important target in detecting OPNA exposure. An albumin adduct with a longer half-life (~21days) does not age easily, making it suitable for forensic retrospective verification. Unlike enzymes that hydrolyze serine, known modified sites are no longer limited to serine in the ChE structure, including tyrosine and lysine residues. The expanded types of modified sites provide more possibilities for the examination of potential biomarkers. An active site tyrosine 411, with high reactivity and good stability on phosphonylated peptide in human serum adducts (HSA), is an internationally recognized biomarker for OPNA exposure, helping identify and confirm agent poisoning, timely diagnosis, and effective treatment [64,65,66]. GB-Y411 adducts were accurately identified in blood samples from actual cases of OPNA poisoning in Syria [67]. Two peptides VRY_411_TKKVPQVST and LVRY_411_TKKVPQVST, labeled Y411, were reported in human plasma analyzed by MALDI-TOF/TOF mass spectrometry after pepsin digestion [65,68].

More potential biomarkers in albumin are still in the exploration stage; some possible targets and characteristic fragment ions were found [69,70]. The potential biomarkers identified usually refer to those which are highly abundant in the MS spectrum and can be modified stably by OPNAs, while the characteristic ion is the ion fragment that appears stably in the cleavage process of protein adducts in the retrospective exploration experiment. For example, Y263 in the peptide Y_263_ICENQDSISSK can be modified stably by six agents (GA, GB, GD, VX, ethyl GA and propyl GA), and related characteristic signals for labeled tyrosine were found, such as O-ethyl methylphospho Tyr immonium of VX modified peptide at m/z 242, methylphospho Tyr immonium at m/z 214.1, belonging to peptide labeled by VX, GB, GF, and unique characteristic ions from phosphonylated peptide by ethyl GA and propyl GA [71]. The common characteristic methylphospho Tyr immonium of OPNAs tyrosine adducts can identify phosphonylated tyrosine, independent of peptides. The characteristic ions on modified GA-lysine peptides were also reported, such as phospho Lys immonium ion at m/z 236.1522, phospho Lys immonium ion-NH_3_ at m/z 219.1257 and phospho Lys immonium ion-NH_3_-C_2_H_4_ at m/z 191.0944, which help to determine common characteristic ions (Lys immonium ion-NH_3_ at m/z 84.0808), or characteristic ions of other OPNA-lysine adducts [72]. Four lysine sites K212, K414, K199, and K351 were modified by diisopropylfluorophosphate, and common specific fragments were displayed at 164.0, 181.2 and 83.8 amu. These reliable characteristic product ions—such as tyrosine and lysine immonium ions—suggest independent phosphonylation modification by the addition of residual groups from OPNAs.

A novel potential biomarker, S419, on the peptide KVPQVSTPTLVESR in HSA was observed; it was modified simultaneously by nine agents and a high mass spectrum response. S419 was phosphonylated by GB and GF, while seven other agents, such as GD, GA and VX, tended to link to K414 [73]. The result emphasized the importance of peptides in providing information regarding OPNA exposure and helped to preliminarily screen possible OPNA varieties. Two serine sites in HSA were labeled by FP-Biotin at S232 on AEFAEVS*K and S287 on S*HCIAEVENDEMPADLPSLAADFVESK, respectively. The product ions of the latter were unreliable due to excessive molecular weight (3546.6 amu). The corresponding characteristic ions from the peptide AEFAEVS*K were described as follows: the parent ion with two protons was at 726.9amu; the product ion from aged FP-biotin released from S232 was at 591.3 amu. However, compared with tyrosine and lysine adducts, the serine adducts identified had several disadvantages, such as poor stability and effortless aging; few characteristic product ions were reported. Some specific ions of protein-independent OPNA adducts were also mined to be used specifically for the identification of OPNAs. For example, Fu et al. [74] revealed the fragmentation pathway of GD adducts with HSA, analyzed by the LC–MS system using an electrospray ionization mode, after trypsin digestion. The authors found three naturally-losing product ions [M+2H-54]^2+^, [M+2H-72]^2+^ and [M+2H-84]^2+^ with high abundance, existing independently in the modified peptides, which did not rely on the properties of the bound protein, suggesting coherent information for GD exposure.

Disulfide adducts, typical and unique to V-type OPNAs, are formed by thiol groups of cysteine residue linked to the thiol group from the departing group of class V agents. Cysteine residue (Cys34), containing free thiol groups in albumin, can form a disulfide adduct with the departing group in the OPNA structure. The LC–MS/HRMS system identified the dipeptide cysteine-proline (Cys-Pro) modified by 2-(diethylamino)-ethylthiol (DEAET) from VX and VR after pronase digestion [50]. The authors also presented a protonated molecule in the MS^2^ spectrum at m/z 350.16 and summarized possible characteristic ions with high intensity at m/z 132.08, 166.07 and 217.06. In addition, other disulfide adducts, such as inverted dipeptide ProCys _448/487_-DPAET with two characteristic product ions at m/z 160.1165 and m/z 217.0652, tripeptide MetProCys_448_-DPAET and AspIleCys_514_-DPAET, were reported successively. Andreas et al. [75] believed the formation pathway of ion fragments from disulfide adducts relies mainly on β-elimination, α-bond cleavage, and disulfide bond cleavage. Additionally, the formation of the disulfide adduct is always accompanied by phosphonylated adducts, emphasizing the inevitable connection between the two adducts. For instance, Andreas et al. [76] observed simultaneously phosphonylated tyrosine Y-VX and disulfide adducts DPAET-CP, labeled by VX, based on a µLC-ESI HRMS/MS method after pronase digestion. Fu et al. [77] postulated that the information from a phosphonylation adduct, accompanied a losing departing group that experienced bonding to cysteine residue by a disulfide bond. This view was confirmed repeatedly. Table 1 shows potential biomarkers with high reactivity and related signals of albumin adduct.

## 5. Conclusions 

This work reviewed various biomarkers in biological samples for retrospective determination of OPNA exposure and approaches to relevant identification. Biomarkers are classified into intact OPNAs, degradation products and protein adducts, based on the existing forms of OPNAs in organisms. The rapid distribution and metabolism in the body of intact OPNAs with high reactivity and poor stability make them difficult to detect. Direct agent identification approaches, such as the competitive ELISA method and enzyme-based microassay, were successfully applied to determine the existence of free OPNAs. However, the premise for applying these methods is to obtain biological samples containing residual OPNAs. In actuality, when the absolute amount of OPNAs is sufficiently low, only degradation products or adducts are detected—not residual agents. To obtain more accurate evidence of OPNA exposure, degradation products and protein adducts have become significant targets for retrospective study after OPNA exposure. Degradation products and adducts in biological samples are usually identified using a GC–MS or LC–MS system. After derivatization, degradation products, polar compounds with low volatility, can be easily captured within 48 h by GC–MS analysis. Unfortunately, prior derivatization is often complicated and time-consuming, and because it forms more by-products, strong background signals and ion source pollution, it may increase the difficulty of analysis. Compared with the GC–MS system, LC-MS analysis for degradation products omits derivatization, except in special cases such as MPA with high polarity and low molecular weight.

Protein adducts with larger detection windows are the most promising targets for retrospective detection of OPNA exposure. However, phosphonylation of the target protein by OPNAs can slightly change the molecular weight, which is difficult to directly capture by mass spectrometry. Thus, two analytical methods are reported: fluoride-induced reactivation and digestive peptide analysis. The fluoride regeneration method usually utilizes a GC or a GC–MS system to analyze various agents bound to AChE, BChE and albumin in biomedical samples. Unfortunately, this method cannot directly distinguish the regenerated agents from the free form and is not suitable for aged protein adducts, which may hinder the regeneration of fluorophosphates. Digestive peptide analysis using the LC–MS system is a common method for protein adducts which successfully avoids these problems. The most common target proteins include AChE in red blood cells, BChE, and albumin in serum or plasma. The nonapeptide from BChE adducts and Y411 adducts in human albumin are recognized biomarkers in retrospective research after OPNA exposure. The characteristic ion fragments in the MS/MS spectrum of the nonapeptide in the BChE adduct provide adequate evidence for determining OPNA exposure and for confirming the agent’s identity. More active sites, potential biomarkers and characteristic ion fragments are constantly discovered, providing ever more effective information for forensic evidence.

At present, degradation products, the nonapeptide FGES_198_AGAAS of BChE adduct, and Y411 in albumin adduct in biomedical samples, are successfully used as biomarkers in actual cases of OPNA exposure. These biomarkers with high reactivity and good stability, and acknowledged classic methods, are repeatedly verified, independent of research institutions, laboratories and research teams. However, more potential biomarkers and innovative research methods are discovered, or proposed by laboratories or research teams, without repeated verification. Even though potential biomarkers are constantly proposed, there are few recognized biomarkers that can be used in actual situations. In fact, the potential biomarkers found may be random due to differences in research methods and operating procedures. Therefore, there is an urgent need to use legitimized methods for screening stable and reliable biomarker candidates from a large number of potential biomarkers. In short, various laboratories experiment on the research of others to discover potential biomarkers and characteristic ions and to pursue the reliability of their results. In addition, future research must focus on improving these methods. Most of the studies on retrospective detection of OPNA exposure are basic research for exploration, which means that the research of potential biomarkers is conducted on the premise that the identity of the OPNA is known. Known agents often make the analysis of biomarkers in the mode with the highest reactivity. However, in actual biomedical samples, unknown OPNAs must operate in the scanning mode of MS, which largely reduces sensitivity. For this reason, mass spectrometry detectors with high resolution, high reactivity and accurate data processing system in the scanning mode, should be further improved.

Retrospective identification of nerve agent biomarkers in biomedical samples is significant in determining OPNA exposure. Effective and reliable biomarkers provide more accurate information—such as the identity of the OPNAs—and more precise treatment. Moreover, retrospective detection of biomarkers after OPNA exposure is of great importance to forensic analysis in actual cases, such as terrorist attacks, assassination and OPNA poisoning, as well as being a dependable basis for chemical accident judgment. Therefore, it is absolutely necessary to systematically summarize biomarkers and research methods in this work.

## Figures and Tables

**Table 1:** Potential biomarkers with high reactivity and related signals of albumin adduct.

Potential Biomarkers with High Reactivity	Other Signals	Sample	Protease	Analytical Method	Agents	References
VRY_411_TKKVPQVSTPTL LVRY_411_TKKVPQVSTPTL	Product ions	Human albumin and plasma	Pepsin	MALDI-TOF MS	GB	[65]
YKFQNALLVRY_411_TKKVPQVSTPTLVE IARRHPY_148_FY_150_APE LLFFAKRY_161_KAAFTE	Any sites	Pure HSA and plasma	Trypsin, pepsin, pronase	MALDI-TOF MS	GA, GB, GD, GF, VX, Vs, VR	[66]
VRY_411_TKKVPQVST LVRY_411_TKKVPQVST		Human plasma	Pepsin	MALDI-TOF/TOF MS	GD	[68]
RY*GRK	Any sites	Albumin of human and mouse	Pepsin	MALDI-TOF-TOF MS	GD	[69]
VX and GD-tyrosines		Plasma of rhesus monkeys	Pepsin	LC–MS-MS SRM mode	GD, VX	[70]
Y_263_ICENQDSISSK	Any sites	HSA	Trypsin	Q Exactive LC-MS/MS	GA, GB, GF, VX, ethyl GA, and propyl GA,	[71]
FPK_224_AEFVEVTK K_524_QTALVELLK AYK_212_AWALVR LDALK_186_EKALISAAQER K_414_VPQVSTPTLVEISR LC*AIPK_79_LR LDAVK_186_EK	Any sites and characteristic ions	Albumin of bovine, leporine, and rat	Trypsin	Orbitrap LC-MS/HRMS	GA	[72]
K_414_VPQVS_419_TPTLVESR	Any sites	HSA	Trypsin	ESI-Q-Orbitrap MS	GA, GB, GD, GF, VX, Vs, VR, MEGA, EEGA	[73]
DEAET-C_34_P	Product ions	Human plasma	Pronase	LC-MS/HR MS	VR, VX	[50]
K_525_QTALVELVK LK_199_CASLQK	Any lysine sites, Product ions for GAs-lysine	HSA, RSA in vitro and in vivo	Trypsin	ESI-Q-Orbitrap MS	GA	[51]
EK_188_ALISAAQER YK_162_AILTECCEAADK	Any sites	Rabbit albumin in vitro and in vivo	Trypsin	nLC-Q-Orbitrap-MS	GD	[78]
DPAET-Cys_34_Pro MetProCys_448_-DPAET AspIleCys_514_-DEAET	Product ions	Human plasma	Pronase	μLC-ESI MS/HRMS	VX, VR, Vs	[75]
PC_34_-DPAET MPC_448_-DPAET DIC_514_-DPAET	Product ions	HSA	Pronase	µLC-ESI MS/HR MS	VX	[76]
DPAET-C_34_P PC_34_-DPAET	Any sites	HSA	Pronase	µLC-ESI HR MS/MS	VX	[79]
